# Engineered Passive Glucose Uptake in *Pseudomonas taiwanensis* VLB120 Increases Resource Efficiency for Bioproduction

**DOI:** 10.1111/1751-7915.70095

**Published:** 2025-01-27

**Authors:** Tobias Schwanemann, Nicolas Krink, Pablo I. Nikel, Benedikt Wynands, Nick Wierckx

**Affiliations:** ^1^ Institute of Bio‐ and Geosciences, IBG‐1: Biotechnology Forschungszentrum Jülich GmbH Jülich Germany; ^2^ The Novo Nordisk Foundation Center for Biosustainability Technical University of Denmark Kongens Lyngby Denmark

**Keywords:** ATP consumption, glucose transport, metabolic engineering, *Pseudomonas*, strain optimization

## Abstract

Glucose is the most abundant monosaccharide and a principal substrate in biotechnological production processes. In *Pseudomonas*, this sugar is either imported directly into the cytosol or first oxidised to gluconate in the periplasm. While gluconate is taken up via a proton‐driven symporter, the import of glucose is mediated by an ABC‐type transporter, and hence both require energy. In this study, we heterologously expressed the energy‐independent glucose facilitator protein (Glf) from *Zymomonas mobilis* to replace the native energy‐demanding glucose transport systems, thereby increasing the metabolic energy efficiency. The implementation of passive glucose uptake in engineered production strains significantly increased product titres and yields of the two different aromatic products, cinnamic acid (+10%–15%) and resveratrol (+26%; 18.1 mg/g) in batch cultures.

## Graphical abstract

In this study, we heterologously expressed the energy‐independent glucose facilitator protein (Glf) from *Zymomonas mobilis* to replace the native energy‐demanding glucose transport systems, thereby increasing the metabolic energy efficiency. Implementation of passive glucose uptake in engineered production strains significantly increased product titres and yields of two different aromatic products.

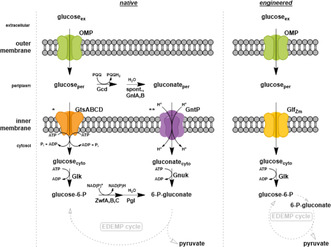

**Abbreviations**
Gcd: glucose dehydrogenase
Glf: glucose facilitator protein
GRC3: genome‐reduced *chassis* strain number 3
MC‐III: malonyl‐CoA *chassis* strain number 3
MC‐IV: malonyl‐CoA *chassis* strain number 4
MSM: mineral salt medium

## 1 Introduction

The uptake of carbohydrates is a fundamental process of microbial life. Especially for the uptake of sugars across biological membranes, a plethora of different sugar utilisation and transport systems have evolved in bacteria (Jeckelmann and Erni 2020) in high dependency on the respective ecological niche. Biotechnological processes usually differ significantly from the microorganism's natural habitats, for example, by high concentrations of carbon sources and a lack of microbial competition. It is thus not surprising that the native sugar uptake and metabolism are not necessarily ideal for the applied bioprocess. In *Pseudomonas*, glucose enters the periplasm from the extracellular space via porins like one of four OprB (Wylie and Worobec 1995). It is subsequently taken up into the cytosol via ATP‐binding cassette (ABC) transporter GtsABCD at the expense of ATP (del Castillo et al. 2007; Thomas and Tampé 2020) or oxidised to gluconate by periplasmic glucose dehydrogenase (GDH, Gcd) and gluconolactonases (Nerke et al. 2024). Periplasmic oxidation of glucose allows pseudomonads to shunt electrons via the pyrroloquinoline quinone (PQQ) cofactor directly into the respiratory chain (An and Moe 2016) and is considered the major route for glucose utilisation in many pseudomonads (Kohlstedt and Wittmann 2019). Periplasmic gluconate can further be oxidised to 2‐ketogluconate by the gluconate 2‐dehydrogenase complex (Gad; PP_3382‐3384) in some pseudomonads (Kohlstedt and Wittmann 2019; Volke et al. 2023). *Pseudomonas taiwanensis* VLB120, a well‐established bioproduction platform, lacks this second periplasmic oxidation. In contrast to GtsABCD, the gluconate transporter GntP/GntT or 2‐ketogluconate transporters KguT both belong to the major facilitator superfamily (MFS) proton symporters (del Castillo et al. 2007) and are thus also energy‐dependent (Lagarde 1977).

**FIGURE 1:**
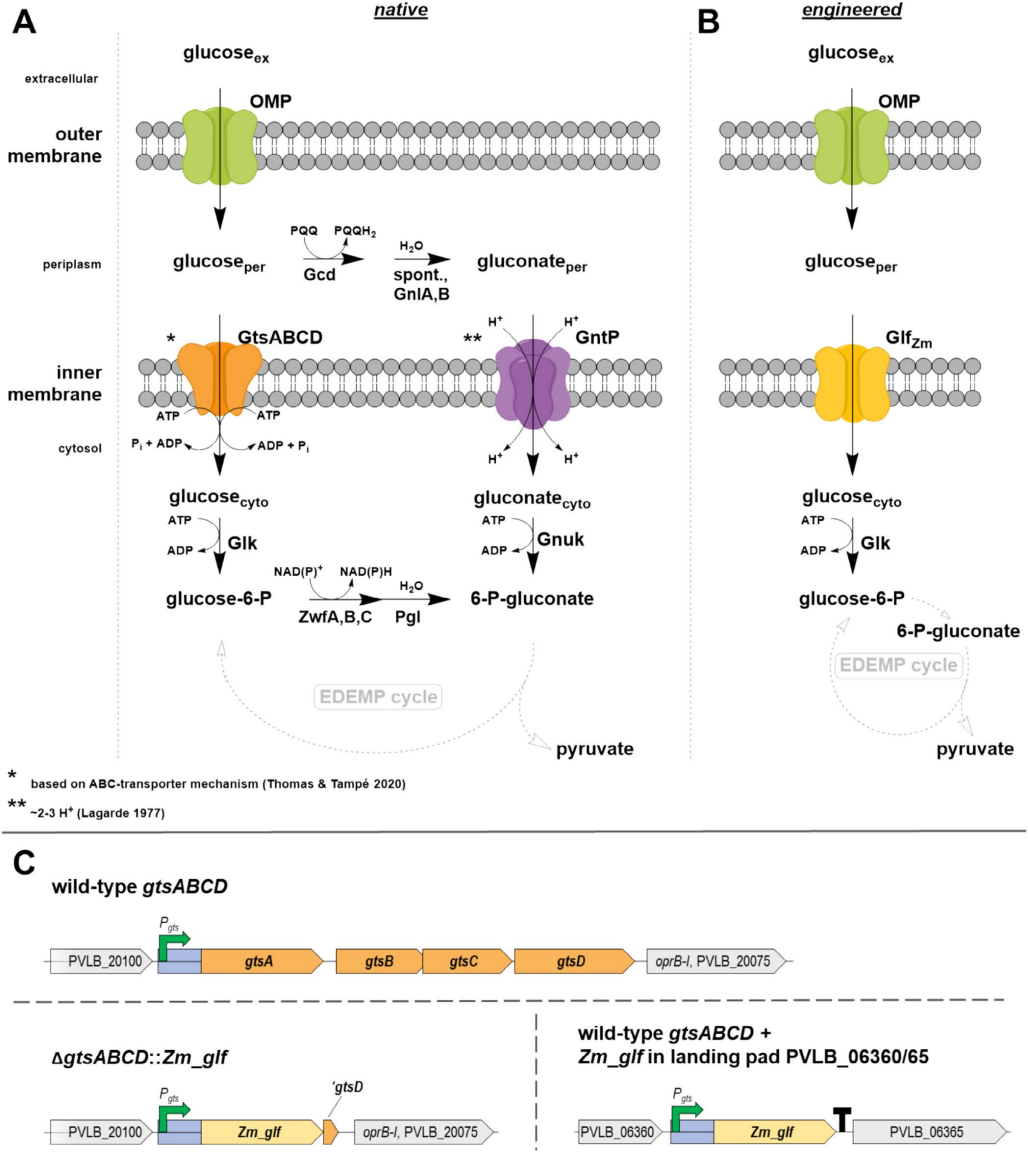
Engineered glucose uptake in *P. taiwanensis* . (A) Overview of native and (B) engineered glucose transporters in *P. taiwanensis* VLB120. Energetic drivers are illustrated according to the mechanism of transport indicated by asterisks. (C) Genetic organisation of glucose uptake‐encoding genes in wild‐type and engineered *P. taiwanensis* VLB120. '*gtsD*, remaining 32 bp of truncated coding sequence of native GtsD; EDEMP cycle, Entner–Doudoroff‐Embden‐Meyerhof‐Parnas‐pentose phosphate cycle; Gcd, PQQ‐dependent glucose dehydrogenase; Glf_Zm_, glucose facilitator protein from *Zymomonas mobilis* ; Glk, glucokinase; Gnl, gluconolactonase; GnuK, d‐gluconate kinase; GtsABCD, glucose ABC transporter; GntP, d‐gluconate transporter; OMP, outer membrane porins; *oprB‐I*, coding sequence of a carbohydrate‐selective porin; Pgl, 6‐phosphogluconolactonase; *P*_*gts*_, presumed promoter region (200 bp upstream of the start codon of *gtsA* PVLB_20095–80); PQQ, pyrroloquinoline quinone; Zwf, Glucose‐6‐phosphate dehydrogenase (Nikel et al. 2015).

After translocation into the cytosol, glucose is phosphorylated by glucokinase (Glk) and subsequently converted by one of the three glucose 6‐phosphate dehydrogenase (Zwf) isoenzymes (Volke, Olavarría, and Nikel 2021) and 6‐phosphogluconolactonase (Pgl) to yield 6‐phosphogluconate, which is an intermediate of the EDEMP cycle (Nikel et al. 2015) (Figure 1). Regulation of glucose uptake and sugar catabolism is controlled *inter alia* by the two‐component system response regulator GltR‐II (PP_1012 in *Pseudomonas putida* KT2440; PVLB_20105 in *P. taiwanensis* VLB120) and the repressor HexR (PP_1021; PVLB_20065) (del Castillo, Duque, and Ramos 2008; Lim et al. 2022; Udaondo et al. 2018). 2‐Keto‐3‐deoxy‐6‐phosphogluconate (KDPG) is one of the effectors of HexR (Kim, Jeon, and Park 2008) whose deletion has been shown to be beneficial for the production of *cis*,*cis*‐muconate in strains lacking Gcd due to the derepression of the intracellular catabolic genes (Bentley et al. 2020; Rorrer et al. 2022). In certain bioprocesses, excessive periplasmic oxidation of glucose to gluconate via gluconolactone is disadvantageous due to the acidification of the culture broth as this can inhibit microbial growth or require titration of base in pH‐controlled cultivations. To avoid massive pH fluctuations or the need of excessive titration of base and acid while simultaneously enabling the application of higher glucose concentration in batch cultivations, the respective *gcd* gene can be deleted and, depending on the metabolic context, this may even lead to improved production, for example, muconic acid, polyhydroxyalkanoates and polyketides to name a few (Bentley et al. 2020; Poblete‐Castro et al. 2013; Schwanemann et al. 2023) or avoid the unwanted bypassing conversion of alternative dimeric carbohydrates (Dvořák and de Lorenzo 2018). However, this deletion may cause detrimental effects because gluconate formation is involved in a wide regulatory network (Volke et al. 2023). Strategies to implement alternative uptake systems may further improve production in strains lacking Gcd. This would be widely applicable in many different cultivation approaches that use glucose as substrate.

Native energy‐driven glucose transport systems are often beneficial in environments with low carbohydrate concentration because they allow uptake with high affinity (Jeckelmann and Erni 2020). This can be a distinct advantage under competitive conditions with limited glucose availability. In an artificial laboratory environment, substrates are usually supplied in concentrations exceeding the physiological K_M_ values of the respective uptake systems by several orders of magnitude. Therefore, the native sugar transport is usually not adapted to bioprocess requirements (Jeckelmann and Erni 2020) and in the case of some pseudomonads, it leads to excessive acidification by gluconate as a carbon sequestration strategy (del Castillo et al. 2007).

An alternative glucose uptake system, specifically interesting for biotechnological applications, can be found in *Zymomonas mobilis* . This bacterium naturally occurs in carbohydrate‐rich environments and is an established facultative anaerobic host for ethanol fermentation with higher ethanol yields than yeasts due to its use of the Entner–Doudoroff pathway and limited biomass formation (Rogers, Lee, and Tribe 1976; Wang et al. 2018; Yang et al. 2016). In *Z. mobilis* , glucose is taken up along the concentration gradient through a glucose facilitator (Glf) protein without the expense of energy (Snoep et al. 1994) unlike the glucose phosphotransferase system of *Escherichia coli* and the ABC glucose transporter of pseudomonads. The facilitated diffusion mediated by Glf_Zm_ enables glucose uptake at a very high specific uptake rate (Fuhrer, Fischer, and Sauer 2005) but with a low reported affinity of K_M_ 1–4 mM (Parker et al. 1995; Weisser et al. 1995). Xylose and fructose (K_M_ 39 mM) are also transported, but with much lower affinities compared to glucose (Kurgan et al. 2021; Weisser et al. 1995). Glf_Zm_ appears as a promising transporter for sugars in an energy‐independent manner that might be favourable in bioprocesses with high metabolic energy demands caused by, for example, osmotic stress or product toxicity. In *E. coli* , replacing the PEP‐consuming phosphotransferase system with Glf_Zm_ increased shikimate production (Yi et al. 2003), while the expression of Glf_Zm_ in *P. putida* enabled the use of alternative carbohydrates (Bujdoš et al. 2023).

In this study, Glf_Zm_ was introduced into several genome‐reduced *chassis* strains (GRC3) of *P. taiwanensis* VLB120. These strains were previously tailored for improved efficiency in bioprocesses by removal of dispensable or disadvantageous cellular features (Wynands et al. 2019), which is expanded in this study by the engineering of glucose uptake. Its impact on production strains was investigated for the biosynthesis of resveratrol and cinnamate, showcasing the applicability of Glf_Zm_ and its benefits in *Pseudomonas* for the production of different molecules from glucose.

## 2 Materials and Methods/Experimental Section

### 2.1 Cultivation Conditions, Media, DNA Techniques

*Escherichia coli* and *Pseudomonas taiwanensis* VLB120 strains were cultured on agar plates (15 g L^−1^) or in LB medium (10 g L^−1^ peptone, 5 g L^−1^ sodium chloride, and 5 g L^−1^ yeast extract) or modified mineral salts medium (MSM) with 3‐fold concentration of buffer components (3 × 22.3 mM K_2_HPO_4_ and 3 × 13.6 mM NaH_2_PO_4_) (Hartmans et al. 1989) at 37°C and 30°C, respectively. Growth experiments started from serial precultures, beginning in LB, followed by an adaptation culture in MSM and the following cultivation under experimental conditions with either 20 or 30 mM glucose. Antibiotics were added if required (50 mg L^−1^ kanamycin sulphate; 20 mg L^−1^ gentamicin sulphate solution; 100 mg L^−1^ ampicillin sodium salt; tetracycline hydrochloride 30 mg L^−1^). For biotransformation experiments for the production of resveratrol, 1 mM *p*‐coumarate was supplemented to the medium. Plasmids were constructed using the NEBuilder HiFi DNA Assembly methodology, and knockout procedures were performed using the I‐SceI system that is based on two consecutive homologous recombination events as described previously (Schwanemann et al. 2023; Wynands et al. 2018). All strains, plasmids and DNA oligonucleotides used in this study are shown in Tables S1, S2 and S3, respectively. The amino acid sequence of the glucose facilitator protein from *Zymomonas mobilis* (Glf_Zm_) used for codon optimization corresponds to UniProt entry P21906 (Table S4). The 200 bp upstream of *gtsABCD* (PVLB_20095–80) was considered as the promoter region of heterologous Glf_Zm_ expression constructs.

Cultivations for the production of cinnamate (Otto et al. 2019) or resveratrol were performed in a 24‐square well plate system Duetz as described previously by Schwanemann et al. (2023).

### 2.2 Analytical Methods

Growth characterisation experiments were performed in 96‐square well plates in the Growth Profiler 960 with respective calibration for conversion of ‘green values’ from pixels of a picture into OD_600_ equivalents.

Determination of the optical density was performed at 600 nm with GE Healthcare Ultrospec 10 device from Fischer Scientific GmbH (Schwerte, Germany).

To determine biomass concentration after 24 h by cell dry weight (CDW) and OD_600_, experiments were executed in 100 mL shake flasks with 11% filling volume, and samples of 10 mL were collected in dried and pre‐weighted glass centrifuge tubes from Glaswarenfabrik Karl Hecht GmbH & Co KG (Sondheim, Germany) that were centrifuged for 20 min at 4000 *g* and washed with 5 mL of a 0.9% (*w*/*v*) sodium chloride solution. After discarding the supernatant, the pellets were dried at 65°C. A respective medium control was processed in parallel.

For the analysis of resveratrol, 1 mL culture broth was extracted with ethyl acetate and processed further in amber glass vials, as described in detail previously (Schwanemann et al. 2023). Cinnamate and *p*‐coumarate were quantified from filtered culture supernatant, and all supplemented precursors and products were analysed by HPLC.

For the detection and quantification of cinnamate, *p*‐coumarate and resveratrol, a 1260 Infinity II HPLC with a 1260 DAD WR (Agilent Technologies) and an ISAspher 100‐5 C18 BDS column (Isera, Düren, Germany) was used, utilising the identical settings and gradients of 0.1% trifluoroacetic acid and acetonitrile as previously for resveratrol analysis (Schwanemann et al. 2023). Cinnamate, *p*‐coumarate and resveratrol were measured at 245, 280, and 310 nm and eluted after 11.54, 7.13, and 9.08 min, respectively.

All experiments were executed in replicates, and significance analysis was performed using 1‐way ANOVA with a confidence interval of *p* < 0.05.

## 3 Results and Discussion

### 3.1 Effect of Glf_Zm_ on the Growth of *P. taiwanensis*

**FIGURE 2:**
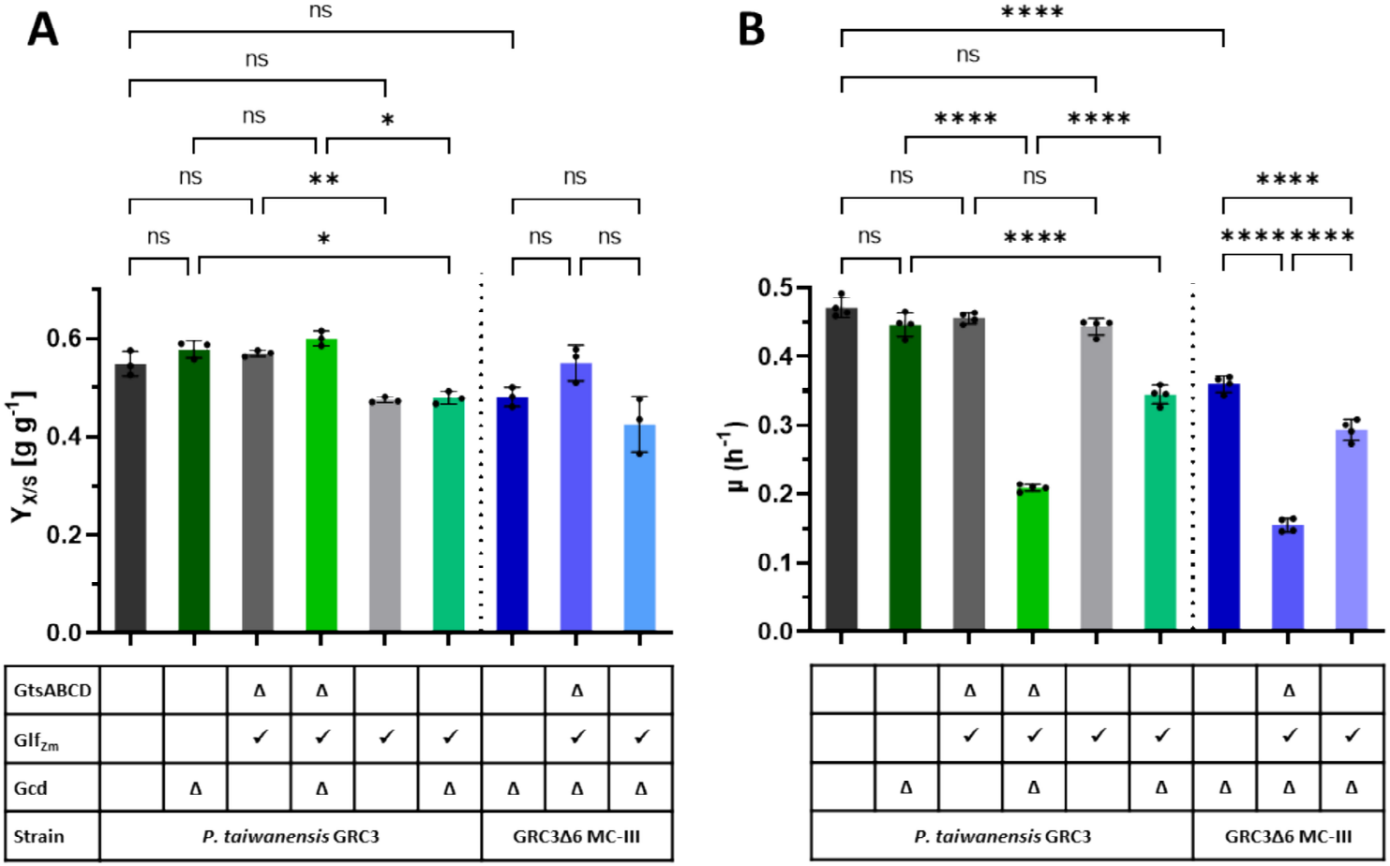
Biomass yields and kinetics of different Glf_Zm_ strains. (A) Final biomass yields (g_biomass_/g_glucose_) resulting from the determined cell dry weight of *P. taiwanensis* VLB120 GRC3, GRC3Δ*gcd* and GRC3Δ6MC‐III with either replaced glucose transporter gene *gtsABCD* by *Zm_glf* indicated by Δ for GtsABCD or with *Zm_glf* expression from landing pad PVLB_06360/65. Grown in shaken‐flask cultures with 30 mM (5.4 g L^−1^) glucose and 3‐fold buffered MSM for 24 h with inoculation of 1% (*v*/*v*) from the adaption culture. (B) Growth rate of the same strains determined in Growth Profiler experiment from OD_600_ equivalent values obtained in 3‐fold buffered MSM with 20 mM glucose (Figure S5). Error bars represent the standard deviation (*n* = 3 in A or *n* = 4 in B). Statistical analysis was made by 1‐way ANOVA (ns, *p* > 0.05; *, *p* < 0.05; **, *p* < 0.01; ****, *p* < 0.0001). Ns, not significant; Y_X/S_, biomass yield; μ, growth rate.

The expression of *Zm_glf*, encoding the glucose facilitator of *Z. mobilis* , in different *P. taiwanensis* GRC3 strains was achieved either through the exchange of glucose transporter genes *gtsABCD* with *Zm_glf* or by the additional expression of *Zm_glf* from a neutral genomic landing pad (PVLB_06360‐65) (Köbbing et al. 2024) (Figures 1B and 2). The same modifications were introduced in GRC3Δ*gcd* and a malonyl‐CoA platform strain (GRC3Δ6 MC‐III) (Schwanemann et al. 2023) which are incapable of periplasmic glucose oxidation to gluconate. To elucidate the effects of these modifications, final biomass concentrations were determined by CDW to calculate biomass yields (Figure 2A) and growth rates were determined by OD_600_ equivalent from Growth Profiler experiments (Figure 2B). The transporter exchange did not alter biomass yields in the GRC3 background. The additional expression of *Zm_glf* even reduced the biomass yield compared to the GRC3 control and the GRC3 strain with the replaced transporter. However, the difference was only significant when compared to the latter. In GRC3Δ*gcd* strain background, a similar trend of final biomass yields was observed for additional Glf_Zm_ expression. The reduced biomass yield might be an effect of limited membrane space (Daddaoua et al. 2017; Nerke et al. 2024) and increased maintenance when *Zm_glf* is additionally expressed from the same promoter as native *gtsABCD*.

Upon expression of *Zm_glf* in strain GRC3Δ6 MC‐III, which was metabolically engineered for increased malonyl‐CoA supply including a *gcd* deficiency (Schwanemann et al. 2023), no significant changes were determined regarding biomass yield, but a reduced growth rate was observed. A trend to yield a high final biomass for the exchanged transporter strain was noted, although the respective control reached a slightly lower final biomass than the GRC3 control strains. That might not be surprising given that strain MC‐III was engineered for bioproduction.

When comparing growth rates of the respective strains (Figures 2B, S5) the deletion of Gcd alone did not decrease the maximal growth rate, but the malonyl‐CoA platform strain, GRC3Δ6 MC‐III, has a 24% decreased growth rate (0.36 h^−1^) compared to the GRC3 (0.47 h^−1^). Strains with intact periplasmic gluconate formation showed no impact on their growth rate compared to the parental strain. However, strains with deleted Gcd and GtsABCD replaced by Glf_Zm_, in which glucose is taken up solely via the heterologous Glf_Zm_, showed a severe growth rate reduction of approximately half. The additional expression of Glf_Zm_ decreased the rate by 23% or 19% for strains lacking only Gcd (GRC3Δ*gcd*) or the malonyl‐CoA platform strain (GRC3Δ6 MC‐III), respectively.

In general, it can be concluded that the glucose uptake systems in *P. taiwanensis* VLB120 can be replaced by Glf_Zm_ with little effect on biomass yield, but the growth rate is significantly affected in engineered strains that rely solely on glucose import through the Glf_Zm_ transporter. Since *Zm*_glf evolved in a different host and under different metabolic conditions at high carbohydrate concentrations, it is likely unbalanced, interfering with the host's cell envelope and expression capabilities, which could be addressed and optimised by adaptive laboratory evolution (ALE). However, the effects of glucose uptake modifications on growth were only tested on non‐producing strains. Exponentially growing *Pseudomonas*, especially genome‐reduced GRC3 with higher energy efficiency (Wynands et al. 2019), is not energy limited (Zobel et al. 2017). Deep engineering for the conversion of glucose to products typically causes growth rate reductions and higher energy demands, and here the use of Glf_Zm_ might be more beneficial.

### 3.2 Effect of Glf_Zm_ on Cinnamate Formation by Engineered *P. taiwanensis*

To test the effect of Glf_Zm_ in *P. taiwanensis* VLB120 aromatics production strains, a high‐yield cinnamate producer was equipped with the alternative glucose uptake system. Strain *P. taiwanensis* GRC3Δ8Δ*pykA*‐tap (Otto et al. 2019) (here called GRC3 PHE) was the basis for the phenylalanine platform strain. When equipped with a phenylalanine ammonia‐lyase (AtPAL2) from *Arabidopsis thaliana* at the genomic Tn7 integration site (*attTn7*::*P*_*14f*_ *‐AtPAL2*), this strain produces cinnamate from glucose with a yield of 23% C‐mol C‐mol^−1^ (Otto et al. 2019).

**FIGURE 3:**
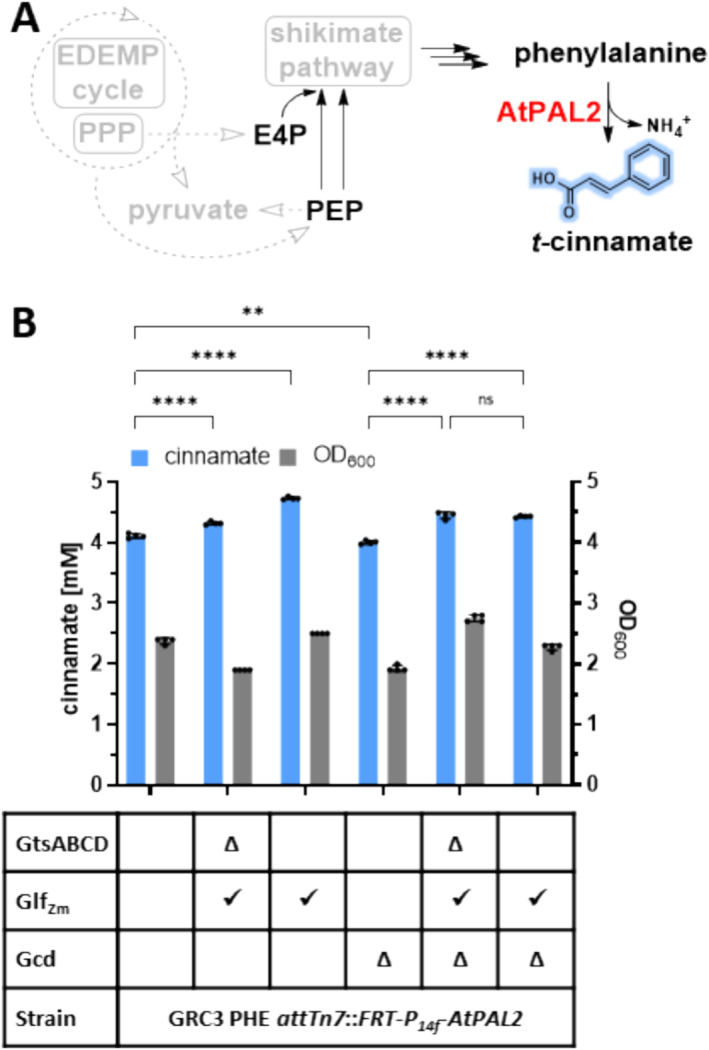
(A) Schematic overview of cinnamate biosynthesis and (B) cinnamate production by different Glf_Zm_ strains. Cinnamate titre and OD_600_ of GRC3 PHE and GRC3 PHE Δ*gcd* with markerless cinnamate production module (*attTn7*::FRT‐*P*_*14f*_ *‐AtPAL2*) with either replaced glucose transporter genes *gtsABCD* by *Zm_glf* or with *Zm_glf* expression from landing pad PVLB_06360/65. Strains were grown in 24‐square deep well plate with 30 mM (5.4 g L^−1^) glucose, 3‐fold buffered MSM, sampled after 115 h in stationary phase. Error bars represent the standard deviation (*n* = 4). Statistical analysis was made by 2‐way ANOVA (ns, *p* > 0.05; **, *p* < 0.01; ****, *p* < 0.0001). E4P, erythrose‐4‐phoasphate; EDEMP cycle, Entner‐Doudoroff‐Embden‐Meyerhof‐Parnas‐pentose phosphate cycle; PEP, phosphoenolpyruvate; PPP, pentose phosphate pathway.

Cinnamate biosynthesis was evaluated in engineered strains featuring either native or modified glucose transport, as well as with and without periplasmic oxidation of glucose to gluconate (Figure 3). In strains with exchanged glucose uptake system, titres were significantly improved by 5% to 4.3 ± 0.03 mM. Further, a 15% increase to 4.7 ± 0.03 mM was observed in the production strain with additional expression of *Zm_glf*. In this strain, the expression driven by the *P*_*gts*_ promoter may compete with native GtsABCD expression and gluconate uptake. Deletion of *gcd* slightly reduced final OD_600_ and had a minor but significant negative effect on cinnamate titres (4.1 ± 0.04 mM for GRC3 PHE, 4.0 ± 0.03 mM GRC3 PHE Δ*gcd*). This relatively minor effect on production would likely be offset in scaled‐up batch cultures through the avoidance of transient acid formation. Without periplasmic Gcd, both expression strategies for Glf_Zm_ improved cinnamate titres to approximately 4.4 mM from 30 mM glucose compared to strain GRC3 PHE Δ*gcd*, which constitutes a significant 10% improvement in production.

### 3.3 Effect of Glf_Zm_ on Resveratrol Formation in *P. taiwanensis*

**FIGURE 4:**
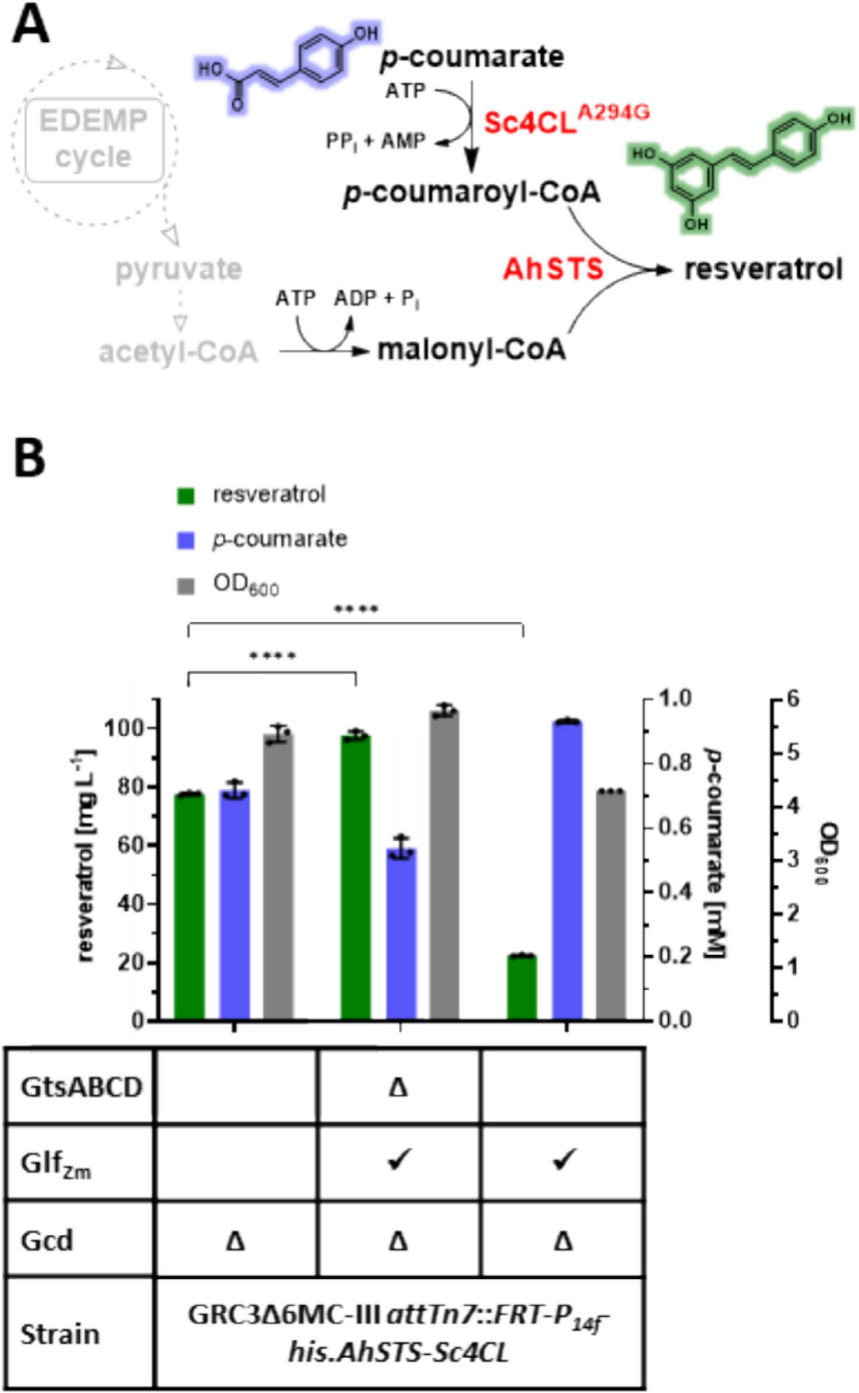
(A) Schematic overview of resveratrol biosynthesis and (B) resveratrol production by different Glf_Zm_ strains. Resveratrol and *p*‐coumarate titre of GRC3Δ6MC‐III with stilbene module (*attTn7*::*FRT‐P*_*14f*_ *‐his.AhSTS‐Sc4CL^A294G^*) with either replaced glucose transporter gene *gtsABCD* by *Zm_glf* or with additional *Zm_glf* expression from landing pad PVLB_06360/65. Grown in 24‐square deep well plate with 30 mM (5.4 g L^−1^) glucose, 3‐fold buffered MSM and 1 mM *p*‐coumarate for 24 h. Error bars represent the standard deviation (*n* = 3). Statistical analysis was made by 2‐way ANOVA (****, *p* < 0.0001). EDEMP cycle, Entner‐Doudoroff‐Embden‐Meyerhof‐Parnas‐pentose phosphate cycle.

High‐yield cinnamate production poses a high drain on central metabolites with a relatively non‐toxic product. In contrast, secondary metabolites are typically produced at lower yields and titres, but their production can pose a higher stress on the cell in terms of metabolic burden. To differentiate between these effects, a malonyl‐CoA platform strain (GRC3Δ6 MC‐III) was evaluated for its ability to produce resveratrol from glucose and *p*‐coumarate (Figure 4) with implemented *Zm_glf* modifications. Resveratrol production was enabled by equipping the strain with the corresponding stilbene production module (*attTn7*::*FRT‐P*_*14f*_ *‐his.AhSTS‐Sc4CL* ^*A294G*^). The GRC3Δ6 MC‐III control with the stilbene module produced 77.6 mg L^−1^ (0.34 mM) resveratrol from 30 mM glucose and 1 mM *p*‐coumarate with 0.72 mM remaining precursor. This resveratrol production is in a similar range to those previously reported (Schwanemann et al. 2023). By exchanging the GtsABCD glucose transporter with Glf_Zm_, resveratrol titres were increased to 97.7 mg L^−1^ (0.43 mM), which represents a 26% improvement and a yield of 18.1 mg_resveratrol_ g_glucose_^−1^. Since this strain background already features a *gcd* knockout, this constitutes a complete replacement of glucose uptake by Glf_Zm_. In contrast to previous reports (Braga et al. 2018), no product loss was observed (Figure S6), with only 0.54 mM *p*‐coumarate remaining. The additional expression of *Zm_glf* in a GtsABCD^+^ background reduced overall biomass and resveratrol titre (Figure 4).

Consequently, GRC3Δ6 MC‐III Δ*gtsABCD*::*Zm_glf* with stilbene production module was identified as a better platform for resveratrol production from *p*‐coumarate than GRC3Δ6 MC‐III in spite of its reduced growth rate (Figure 2) and thus strain *P. taiwanensis* GRC3Δ6MC‐III Δ*gtsABCD*::*Zm_glf* is hereafter called GRC3Δ6MC‐IV (generation No. 4 of the malonyl‐CoA platform strain).

## 4 Conclusion

In this study, we investigated the impact of modified glucose uptake by a passive facilitator in *P. taiwanensis* VLB120 GRC3 and derivatives thereof. Final biomass yields of GRC3 and Gcd‐deficient variants were not affected significantly when *gtsABCD* was replaced by Glf_Zm_. Additional expression of Glf_Zm_ did not necessarily lead to improved growth on glucose or production, indicating that glucose uptake is not limited by transport *per se* or that membrane stress from the additional transport protein outweighed any potential benefit. In the absence of production, the resource‐efficient passive glucose uptake came at the cost of reduced growth rates when both native glucose uptake and periplasmic oxidation were eliminated. However, this replacement boosted bioproduction of value‐added aromatic molecules from different primary pathways in strains that already were highly optimised, highlighting the strategy's applicability, especially under demanding biotechnological conditions. The enhancement was observed not only in the synthesis of cinnamate (10% increase), a shikimate pathway‐derived product, but also in resveratrol production (26% increase), which is derived from malonyl‐CoA and supplemented *p*‐coumarate. Hereby Glf_Zm_ can demonstrate its benefits in the absence of Gcd when the used host relies on cytosolic glucose metabolism, which is used in several bioprocess setups to ensure pH stability. However, the implementation of Glf_Zm_ in production strains with native glucose oxidation can be improved as well, as demonstrated for cinnamate formation. This straightforward engineering of the strain could boost future efforts in optimising *Pseudomonas* for production applications.

## Author Contributions

**Tobias Schwanemann:** conceptualization, methodology, validation, formal analysis, investigation, funding acquisition, writing – original draft, visualization. **Nicolas Krink:** conceptualization, writing – review and editing, supervision. **Pablo I. Nikel:** conceptualization, resources. **Benedikt Wynands:** validation, writing – review and editing, supervision. **Nick Wierckx:** conceptualization, validation, resources, writing – review and editing, supervision, funding acquisition, project administration.

## Conflicts of Interest

The authors declare no conflicts of interest.

## Supporting information


Data S1.

## Data Availability

The data that support the findings of this study are available from the corresponding author upon reasonable request.

## References

[mbt270095-bib-0001] An, R. , and L. A. Moe . 2016. “Regulation of Pyrroloquinoline Quinone‐Dependent Glucose Dehydrogenase Activity in the Model Rhizosphere‐Dwelling Bacterium *Pseudomonas putida* KT2440.” Applied and Environmental Microbiology 82, no. 16: 4955–4964. 10.1128/AEM.00813-16. 27287323 PMC4968544

[mbt270095-bib-0002] Bentley, G. J. , N. Narayanan , R. K. Jha , et al. 2020. “Engineering Glucose Metabolism for Enhanced Muconic Acid Production in *Pseudomonas putida* KT2440.” Metabolic Engineering 59: 64–75. 10.1016/j.ymben.2020.01.001. 31931111

[mbt270095-bib-0003] Braga, A. , J. Oliveira , R. Silva , et al. 2018. “Impact of the Cultivation Strategy on Resveratrol Production From Glucose in Engineered *Corynebacterium glutamicum* .” Journal of Biotechnology 265, no. July 2017: 70–75. 10.1016/j.jbiotec.2017.11.006. 29141192

[mbt270095-bib-0004] Bujdoš, D. , B. Popelářová , D. C. Volke , P. I. Nikel , N. Sonnenschein , and P. Dvořák . 2023. “Engineering of *Pseudomonas putida* for Accelerated Co‐Utilization of Glucose and Cellobiose Yields Aerobic Overproduction of Pyruvate Explained by an Upgraded Metabolic Model.” Metabolic Engineering 75: 29–46. 10.1016/j.ymben.2022.10.011. 36343876

[mbt270095-bib-0005] Daddaoua, A. , A. Corral‐Lugo , J. L. Ramos , and T. Krell . 2017. “Identification of GntR as Regulator of the Glucose Metabolism in *Pseudomonas aeruginosa* .” Environmental Microbiology 19, no. 9: 3721–3733. 10.1111/1462-2920.13871. 28752954

[mbt270095-bib-0006] del Castillo, T. , E. Duque , and J. L. Ramos . 2008. “A Set of Activators and Repressors Control Peripheral Glucose Pathways in *Pseudomonas putida* to Yield a Common Central Intermediate.” Journal of Bacteriology 190, no. 7: 2331–2339. 10.1128/JB.01726-07. 18245293 PMC2293218

[mbt270095-bib-0007] del Castillo, T. , J. L. Ramos , J. J. Rodríguez‐Herva , T. Fuhrer , U. Sauer , and E. Duque . 2007. “Convergent Peripheral Pathways Catalyze Initial Glucose Catabolism in *Pseudomonas putida* : Genomic and Flux Analysis.” Journal of Bacteriology 189, no. 14: 5142–5152. 10.1128/JB.00203-07. 17483213 PMC1951859

[mbt270095-bib-0008] Dvořák, P. , and V. de Lorenzo . 2018. “Refactoring the Upper Sugar Metabolism of *Pseudomonas putida* for Co‐Utilization of Cellobiose, Xylose, and Glucose.” Metabolic Engineering 48: 94–108. 10.1016/j.ymben.2018.05.019. 29864584

[mbt270095-bib-0009] Fuhrer, T. , E. Fischer , and U. Sauer . 2005. “Experimental Identification and Quantification of Glucose Metabolism in Seven Bacterial Species.” Journal of Bacteriology 187, no. 5: 1581–1590. 10.1128/JB.187.5.1581-1590.2005. 15716428 PMC1064017

[mbt270095-bib-0010] Hartmans, S. , J. P. Smits , M. J. Van der Werf , F. Volkering , and J. A. M. De Bont . 1989. “Metabolism of Styrene Oxide and 2‐Phenylethanol in the Styrene‐Degrading *Xanthobacter* Strain 124X.” Applied and Environmental Microbiology 55, no. 11: 2850–2855. 10.1128/aem.55.11.2850-2855.1989. 16348047 PMC203180

[mbt270095-bib-0011] Jeckelmann, J. M. , and B. Erni . 2020. “Transporters of Glucose and Other Carbohydrates in Bacteria.” Pflügers Archiv ‐ European Journal of Physiology 472, no. 9: 1129–1153. 10.1007/s00424-020-02379-0. 32372286

[mbt270095-bib-0012] Kim, J. , C. O. Jeon , and W. Park . 2008. “Dual Regulation of Zwf‐1 by Both 2‐Keto‐3‐Deoxy‐6‐Phosphogluconate and Oxidative Stress in *Pseudomonas putida* .” Microbiology 154, no. 12: 3905–3916. 10.1099/mic.0.2008/020362-0. 19047757

[mbt270095-bib-0013] Köbbing, S. , T. Lechtenberg , B. Wynands , L. M. Blank , and N. Wierckx . 2024. “Reliable Genomic Integration Sites in *Pseudomonas putida* Identified by Two‐Dimensional Transcriptome Analysis.” ACS Synthetic Biology 13: 2060–2072. 10.1021/acssynbio.3c00747. 38968167 PMC11264328

[mbt270095-bib-0014] Kohlstedt, M. , and C. Wittmann . 2019. “GC‐MS‐Based 13 C Metabolic Flux Analysis Resolves the Parallel and Cyclic Glucose Metabolism of *Pseudomonas putida* KT2440 and *Pseudomonas aeruginosa* PAO1.” Metabolic Engineering 54: 35–53. 10.1016/j.ymben.2019.01.008. 30831266

[mbt270095-bib-0015] Kurgan, G. , M. Onyeabor , S. C. Holland , et al. 2021. “Directed Evolution of *Zymomonas mobilis* Sugar Facilitator Glf to Overcome Glucose Inhibition.” Journal of Industrial Microbiology and Biotechnology 49: kuab066. 10.1093/jimb/kuab066. PMC9118996 34529081

[mbt270095-bib-0016] Lagarde, A. 1977. “Evidence for an Electrogenic 3‐Deoxy‐2‐Oxo‐d‐Gluconate–Proton Co‐Transport Driven by the Protonmotive Force in *Escherichia coli* K12.” Biochemical Journal 168, no. 2: 211–221. 10.1042/BJ1680211. 23116 PMC1183754

[mbt270095-bib-0017] Lim, H. G. , K. Rychel , A. V. Sastry , et al. 2022. “Machine‐Learning From *Pseudomonas putida* Transcriptomes Reveals Its Transcriptional Regulatory Network.” Metabolic Engineering 72: 297–310. 10.1016/j.ymben.2022.04.004. 35489688

[mbt270095-bib-0018] Nerke, P. , J. Korb , F. Haala , G. Hubmann , and S. Lütz . 2024. “Metabolic Bottlenecks of *Pseudomonas taiwanensis* VLB120 During Growth on D‐Xylose via the Weimberg Pathway.” Metabolic Engineering Communications 18: e00241. 10.1016/j.mec.2024.e00241. 39021639 PMC11252243

[mbt270095-bib-0019] Nikel, P. I. , M. Chavarría , T. Fuhrer , U. Sauer , and V. De Lorenzo . 2015. “ *Pseudomonas putida* KT2440 Strain Metabolizes Glucose Through a Cycle Formed by Enzymes of the Entner‐Doudoroff, Embden‐Meyerhof‐Parnas, and Pentose Phosphate Pathways.” Journal of Biological Chemistry 290, no. 43: 25920–25932. 10.1074/jbc.M115.687749. 26350459 PMC4646247

[mbt270095-bib-0020] Otto, M. , B. Wynands , C. Lenzen , M. Filbig , L. M. Blank , and N. Wierckx . 2019. “Rational Engineering of Phenylalanine Accumulation in *Pseudomonas taiwanensis* to Enable High‐Yield Production of Trans‐Cinnamate.” Frontiers in Bioengineering and Biotechnology 7: 312. 10.3389/fbioe.2019.00312. 31824929 PMC6882275

[mbt270095-bib-0021] Parker, C. , W. O. Barnelp , J. L. Snoep , L. O. Ingram , and T. Conway . 1995. “Characterization of the *Zymomonas mobilis* .” Molecular Microbiology 15, no. 5: 795–802. 7596282 10.1111/j.1365-2958.1995.tb02350.x

[mbt270095-bib-0022] Poblete‐Castro, I. , D. Binger , A. Rodrigues , J. Becker , V. A. P. Martins Dos Santos , and C. Wittmann . 2013. “In‐Silico‐Driven Metabolic Engineering of *Pseudomonas putida* for Enhanced Production of Poly‐Hydroxyalkanoates.” Metabolic Engineering 15, no. 1: 113–123. 10.1016/j.ymben.2012.10.004. 23164576

[mbt270095-bib-0023] Rogers, P. L. , K. J. Lee , and D. E. Tribe . 1976. “Kinetics of Alcohol Production by *Zymomonas mobilis* at High Sugar Concentrations.” Biotechnology Letters 1: 165–170.

[mbt270095-bib-0024] Rorrer, N. A. , F. Sandra , B. C. Knott , et al. 2022. “Production of β‐Ketoadipic Acid From Glucose in *Pseudomonas putida* KT2440 for Use in Performance‐Advantaged Nylons. Cell Reports Physical.” Science 3, no. 4: 100840. 10.1016/j.xcrp.2022.100840.

[mbt270095-bib-0025] Schwanemann, T. , M. Otto , B. Wynands , J. Marienhagen , and N. Wierckx . 2023. “A *Pseudomonas taiwanensis* Malonyl‐CoA Platform Strain for Polyketide Synthesis.” Metabolic Engineering 77, no. February: 219–230. 10.1016/j.ymben.2023.04.001. 37031949

[mbt270095-bib-0026] Snoep, J. L. , N. Arfman , L. P. Yomano , R. K. Fliege , T. Conway , and L. O. Ingram . 1994. “Reconstitution of Glucose Uptake and Phosphorylation in a Glucose‐Negative Mutant of *Escherichia coli* by Using *Zymomonas mobilis* Genes Encoding the Glucose Facilitator Protein and Glucokinase.” Journal of Bacteriology 176, no. 7: 2133–2135. 10.1128/jb.176.7.2133-2135.1994. 8144485 PMC205325

[mbt270095-bib-0027] Thomas, C. , and R. Tampé . 2020. “Structural and Mechanistic Principles of ABC Transporters.” Annual Review of Biochemistry 89: 605–636. 10.1146/annurev-biochem-011520-105201. 32569521

[mbt270095-bib-0028] Udaondo, Z. , J. L. Ramos , A. Segura , T. Krell , and A. Daddaoua . 2018. “Regulation of Carbohydrate Degradation Pathways in *Pseudomonas* Involves a Versatile Set of Transcriptional Regulators.” Microbial Biotechnology 11, no. 3: 442–454. 10.1111/1751-7915.13263. 29607620 PMC5902321

[mbt270095-bib-0029] Volke, D. C. , N. Gurdo , R. Milanesi , and P. I. Nikel . 2023. “Time‐Resolved, Deuterium‐Based Fluxomics Uncovers the Hierarchy and Dynamics of Sugar Processing by *Pseudomonas putida* .” Metabolic Engineering 79: 159–172. 10.1016/j.ymben.2023.07.004. 37454792

[mbt270095-bib-0030] Volke, D. C. , K. Olavarría , and P. I. Nikel . 2021. “Cofactor Specificity of Glucose‐6‐Phosphate Dehydrogenase Isozymes in *Pseudomonas putida* Reveals a General Principle Underlying Glycolytic Strategies in Bacteria.” MSystems 6, no. 2: msystems.00014‐21. 10.1128/msystems.00014-21. PMC8546961 33727391

[mbt270095-bib-0031] Wang, X. , Q. He , Y. Yang , et al. 2018. “Advances and Prospects in Metabolic Engineering of *Zymomonas mobilis* .” Metabolic Engineering 50: 57–73. 10.1016/j.ymben.2018.04.001. 29627506

[mbt270095-bib-0032] Weisser, P. , R. Krämer , H. Sahm , and G. A. Sprenger . 1995. “Functional Expression of the Glucose Transporter of *Zymomonas mobilis* Leads to Restoration of Glucose and Fructose Uptake in *Escherichia coli* Mutants and Provides Evidence for Its Facilitator Action.” Journal of Bacteriology 177, no. 11: 3351–3354. 7768841 10.1128/jb.177.11.3351-3354.1995 PMC177034

[mbt270095-bib-0033] Wylie, J. L. , and E. A. Worobec . 1995. “The OprB Porin Plays a Central Role in Carbohydrate Uptake in *Pseudomonas aeruginosa* .” Journal of Bacteriology 177, no. 11: 3021–3026. 10.1128/jb.177.11.3021-3026.1995. 7768797 PMC176988

[mbt270095-bib-0034] Wynands, B. , C. Lenzen , M. Otto , F. Koch , L. M. Blank , and N. Wierckx . 2018. “Metabolic Engineering of *Pseudomonas taiwanensis* VLB120 With Minimal Genomic Modifications for High‐Yield Phenol Production.” Metabolic Engineering 47: 121–133. 10.1016/j.ymben.2018.03.011. 29548982

[mbt270095-bib-0035] Wynands, B. , M. Otto , N. Runge , et al. 2019. “Streamlined *Pseudomonas taiwanensis* VLB120 Chassis Strains With Improved Bioprocess Features.” ACS Synthetic Biology 8, no. 9: 2036–2050. 10.1021/acssynbio.9b00108. 31465206

[mbt270095-bib-0036] Yang, S. , Q. Fei , Y. Zhang , et al. 2016. “ *Zymomonas mobilis* As a Model System for Production of Biofuels and Biochemicals.” Microbial Biotechnology 9, no. 6: 699–717. 10.1111/1751-7915.12408. 27629544 PMC5072187

[mbt270095-bib-0037] Yi, J. , K. M. Draths , K. Li , and J. W. Frost . 2003. “Altered Glucose Transport and Shikimate Pathway Product Yields in *E. coli* .” Biotechnology Progress 19, no. 5: 1450–1459. 10.1021/bp0340584. 14524706

[mbt270095-bib-0038] Zobel, S. , J. Kuepper , B. Ebert , N. Wierckx , and L. M. Blank . 2017. “Metabolic Response of *Pseudomonas putida* to Increased NADH Regeneration Rates.” Engineering in Life Sciences 17, no. 1: 47–57. 10.1002/elsc.201600072. 32624728 PMC6999266

