# Supplementary material for: Engineered Passive Glucose Uptake in Pseudomonas taiwanensis VLB120 Increases Resource Efficiency for Bioproduction
Source: Microb Biotechnol. 2025 Jan 27;18(1):e70095. doi: 10.1111/1751-7915.70095 (PMC11772102; doi:10.1111/1751-7915.70095)
Supplement: Supplementary file 1 — Data S1. [file MBT2-18-e70095-s001.docx]

# Supplementary material to article “Engineered passive glucose uptake in *Pseudomonas taiwanensis* VLB120 increases resource efficiency for bioproduction”

Tobias Schwanemann^a^, Nicolas Krink^b^, Pablo I. Nikel^b^, Benedikt Wynands^a^ and Nick Wierckx^a‡^

^a^ Institute of Bio- and Geosciences, IBG-1: Biotechnology, Forschungszentrum Jülich GmbH, 52425 Jülich, Germany

^b^ *The Novo Nordisk Foundation Center for Biosustainability, Technical University of Denmark, Kongens Lyngby, Denmark*

^‡^ corresponding author**:**

Nick Wierckx, Institute of Bio- and Geosciences, IBG-1: Biotechnology, Forschungszentrum Jülich, Wilhelm-Johnen-Straße, 52425 Jülich, Germany. e-mail: n.wierckx@fz-juelich.de

Table of Contents

[Supplementary material to article “Engineered passive glucose uptake in *Pseudomonas taiwanensis* VLB120 for increased resource efficiency in bioproduction” 1](#_Toc176691681)

[**Table S1:** Bacterial strains used in this study 3](#_Toc176691682)

[**Table S2:** Plasmids used in this study 5](#_Toc176691683)

[**Table S3:** Oligonucleotides used in this study 7](#_Toc176691684)

[**Table S4** Coding DNA fragments 8](#_Toc176691685)

[**Figure S5**: 9](#_Toc176691686)

[**Figure S6**: 10](#_Toc176691687)

[References 11](#_Toc176691688)

## **Table S1:** Bacterial strains used in this study

| **Strains** | **Relevant characteristics** | **Reference and strain No.** |
| --- | --- | --- |
| ***Escherichia coli*** |  |  |
| HB101 pRK2013 | HB101 with pRK2013 | Ditta et al. (1980); MiKat #22 |
| PIR2 | *F^−^* ∆*lac169* *rpoS(Am)* *robA1 creC510 hsdR514 endA* *recA1 uidA(*∆*MluI)*::*pir*; host for *oriV(R6K)* plasmids | Thermo Fischer Scientific |
| DH5α λ*pir* | λ*pir* lysogen of DH5α; host for *oriV(R6K)* plasmids | Víctor de Lorenzo lab |
| DH5α λ*pir* pTNS1 | DH5α λ*pir* with pTNS1 | Choi et al. (2005); MiKat #24 |
| DH5α pSW-2 | DH5α with pSW-2 | Martínez-García & de Lorenzo (2011); MiKat #27 |
| ***Pseudomonas taiwanensis*** |  |  |
| GRC3 | Genome-reduced-chassis strain; Δprophage1/2::*ttgVWGHI* | Wynands et al. (2019); MiKat #5 |
| GRC3 PVLB_06360/65::*P_gts_*-*Zm_glf* | GRC3 with additional Glf_Zm_ | This study; MiKat #1709 |
| GRC3 ∆*gtsABCD*::*Zm_glf* | GRC3 with exchanged glucose transporter GtsABCD by Glf_Zm_ | This study; MiKat #1706 |
| GRC3 Δ*gcd* | GRC3 with deleted glucose dehydrogenase (PVLB_05240) | This study; MiKat #684 |
| GRC3 Δ*gcd* PVLB_06360/65::*P_gts_-Zm_glf* | GRC3 Δgcd with additional Glf_Zm_ | This study; MiKat #1710 |
| GRC3 Δ*gcd* ∆*gtsABCD*::*Zm_glf* | GRC3 Δgcd with exchanged glucose transporter GtsABCD by Glf_Zm_ | This study; MiKat #1707 |
| GRC3Δ6 MC-III | Malonyl-CoA platform strain No. 3 | (Schwanemann et al., 2023); MiKat #1009 |
| GRC3Δ6 MC-III PVLB_06360/65::*P_gts_*-Zm_glf | GRC3Δ6 MC-III with additional Glf_Zm_ | This study; MiKat #1654 |
| GRC3Δ6 MC-III ∆*gtsABCD*::*Zm*_*glf* (GRC3Δ6 MC-IV) | GRC3Δ6 MC-III with exchanged glucose transporter GtsABCD by Glf_Zm_, Malonyl-CoA platform strain No. 4 (GRC3Δ6 MC-IV) | This study; MiKat #1618 |
| GRC3Δ6 MC-III *attTn7*::*FRT-P14f-his.AhSTS-Sc4CL^A294G^* | Malonyl-CoA platform strain No. 3 with stilbene module | This study; MiKat #1722 |
| GRC3Δ6 MC-III PVLB_06360/65::*P_gts_*-*Zm*_*glf* *attTn7*::*FRT-P14f-his.AhSTS-Sc4CL^A294G^* | GRC3Δ6 MC-III with additional Glf_Zm_ with stilbene module | This study; MiKat #1813 |
| GRC3Δ6 MC-III ∆*gtsABCD*::*Zm*_*glf* *attTn7*::*FRT-P14f-his.AhSTS-Sc4CL^A294G^* | GRC3Δ6 MC-III with exchanged glucose transporter gtsABCD by glf (GRC3Δ6 MC-IV) with stilbene module | This study; MiKat #1724 |
| GRC3 ∆8-tap-∆pykA (GRC3 PHE) | Phenylalanine platform strain | (Otto et al., 2019); MiKat #74 |
| GRC3 PHE *attTn7*::*P14f-AtPAL2* | Cinnamate producer based on GRC3 PHE, Gm^R^ | (Otto et al., 2019); MiKat #384 |
| GRC3 PHE *attTn7*::*FRT-P14f-AtPAL2* | Cinnamate producer based on GRC3 PHE, without antibiotic resistance | This study, MiKat #1746 |
| GRC3 PHE PVLB_06360/65::*P_gts_*-*Zm_glf* | GRC3 PHE with additional Glf_Zm_ | This study; MiKat #1711 |
| GRC3 PHE PVLB_06360/65::*P_gts_*-*Zm_glf attTn7*::*P14f-AtPAL2* | With cinnamate production module, Gm^R^ | This study, MiKat #1720 |
| GRC3 PHE PVLB_06360/65::*P_gts_*-*Zm_glf* *attTn7*::*FRT-P14f-AtPAL2* | With cinnamate production module, without antibiotic resistance | This study, MiKat #1748 |
| GRC3 PHE ∆*gtsABCD*::*Zm_glf* | GRC3 PHE with exchanged glucose transporter GtsABCD by Glf_Zm_ | This study; MiKat #1708 |
| GRC3 PHE ∆*gtsABCD*::*Zm_glf* *attTn7*::*P14f-AtPAL2* | With cinnamate production module, Gm^R^ | This study; MiKat #1719 |
| GRC3 PHE ∆*gtsABCD*::*Zm_glf* *attTn7*::*FRT*-*P14f-AtPAL2* | With cinnamate production module, without antibiotic resistance | This study, MiKat #1747 |
| GRC3 PHE ∆*gcd* | Phenylalanine platform strain with deleted *gcd* (PVLB_05240) | (Schwanemann et al., 2023); MiKat#339 |
| GRC3 PHE ∆*gcd attTn7*::*P14f-AtPAL2* | With cinnamate production module, Gm^R^ | This study, MiKat #2298 |
| GRC3 PHE ∆*gcd* PVLB_06360/65::*P_gts_*-*Zm_glf* | GRC3 PHE ∆*gcd* with additional Glf_Zm_ | This study; MiKat #2150 |
| GRC3 PHE ∆*gcd* PVLB_06360/65::*P_gts_*-*Zm_glf attTn7*::*P14f-AtPAL2* | With cinnamate production module, Gm^R^ | This study, MiKat #2299 |
| GRC3 PHE ∆*gcd* ∆*gtsABCD*::*Zm_glf* | GRC3 PHE ∆*gcd* with exchanged glucose transporter GtsABCD by Glf_Zm_ | This study; MiKat #2204 |
| GRC3 PHE ∆*gcd* ∆*gtsABCD*::*Zm_glf attTn7*::*P14f-AtPAL2* | With cinnamate production module, Gm^R^ | This study, MiKat #2300 |

## **Table S2:** Plasmids used in this study

| **Plasmid** | **Relevant characteristics** | **HiFi assembly note** | **Reference & No.** |
| --- | --- | --- | --- |
| pTNS1 | Amp^R^, *oriV(R6K)*, TnSABC+D operon | - | Choi et al. (2005) |
| pBBFLP | plasmid for antibiotic markers excision in *P. putida* strains; Tc^R^, *oriV*(pBBR1) *oriT*(RK2) mob^+^ λP_R_::FLP λ(cI857) *sacB* *tet* | - | De Las Heras et al. (2008) |
| pEMG | Km^R^, *oriV(R6K)*, *oriT*, *traJ*, *lacZα*-MCS flanked by two I-SceI restriction sites | - | Martínez-García & de Lorenzo (2011) |
| pSNW2 | Km^R^, *oriV(R6K)*, *oriT*, *traJ*, *lacZα*-MCS flanked by two I-SceI restriction sites, *P_14g_*-BCD2→*msfGFP* | - | Volke et al. (2020, 2021)  plasmid #142 |
| pSW-2 | Gm^R^, *oriV(RK2), oriT, xylS, Pm→* *I-SceI* | - | Martínez-García & de Lorenzo (2011) |
| pSEVA6213S | Gm^R^, *oriV(RK2)*, *P_EM7_*→*I-SceI*; | - | Wirth et al. (2020) |
| pEMG-gcd | Deletion vector for glucose dehydrogenase *gcd* (PVLB_05240) | - | Wynands unpublished, Plasmid #290 |
| pSNW2-Ex-Pro-J23108-PP3303-LP_02480 | Exchange vector for *P_EM7_* in GRC3Δ6 MC-III by *P_J23108_* and BCD10 | Fragment TS-280/TS281 from pSNW2; Fragments TS-310/ TS‑311 and TS-304/TS-305 from GRC3Δ6 MC-II genome; Fragment TS‑309/TS-307 from pSEVA62J23108-BCD10-GFP | Plasmid #515 |
| pSNW2-Ex-gtsABCD-glf KT2440 | Exchange vector for glucose transporter *gtsABCD* with *Zm_glf* in *P. putida* KT2440 | Fragment TS-280/TS281 from pSNW2; Fragments TS‑282/TS‑283 and TS-286/TS-287 from KT2440-genome; Fragment TS‑284/TS-285 from *glf*-template | plasmid #511 |
| pSNW2-Ex-gtsABCD-glf VLB120 | Exchange vector for glucose transporter *gtsABCD* with *Zm_glf* in *P. taiwanensis* VLB120 | Fragment TS-280/TS281 from pSNW2; Fragments TS‑288/TS‑289 and TS-290/TS-291 from VLB120-genome; Fragment TS‑284/TS-285 from *glf*-template | plasmid #512 |
| pSNW2-LP_PVLB06360-65-Pgts-Zm_glf for VLB120 | Insertion of *Zm_glf* into landing pad PVLB_06360/65 in VLB120 with *P_gts_* promoter | Fragment TS-280/TS281 from pSNW2; Fragments TS‑337/TS‑338 and TS-343/TS-344 from VLB120-genome; Fragment TS‑339/TS-340 from MC-III Δ*gtsABCD*::*glf* genome; Fragment TS-341/TS-342 from GRC3Δ6 MC-III genome | plasmid #513 |
| pSNW2-LP_PP1738-Pgts-Zm_glf for KT2440 | Insertion of *Zm_glf* into landing pad PP_1738 in KT2440 with *P_gts_* promoter | Fragment TS-280/TS281 from pSNW2; Fragments TS‑329/TS‑330 and TS-335/TS-336 from VLB120-genome; Fragment TS‑331/TS-332 from SEM11Δ2-glf genome; Fragment TS-333/TS-334 from GRC3Δ6 MC-III genome | plasmid #514 |
| pBG14f-AtPAL2 | cinnamate synthesis module for Tn7 integration | - | (Otto et al., 2019) |
| pBG14f_FRT_Kan | Km^R^ flanked by FRT sites, *oriV(R6K)*, *oriT*, mini-Tn7 transposon delivery vector, *P_14f_(BCD2)*→*msfgfp* | - | Ackermann et al. (2021) |
| pBG14f_Km_FRT_AtPAL2 | Recyclable cinnamate production module for Tn7 integration | Fragment TS-106/TS-019 from pBG14f-Kan-FRT, | Lechtenberg unpublished,  Plasmid #368 |
| pBG14f_Km_FRT_his.AhSTS-Sc4CL^A294G^ | Recyclable stilbene synthesis module with his-tag for Tn7 integration | Fragment TS-368/TS-369 from pBG14f_Km_FRT_his.AhSTS-Sc4CL^A294G^-AtPAL2 (plasmid#229) | This study  Plasmid #537 |

## **Table S3:** Oligonucleotides used in this study

Shown are their name, sequence, and description. Oligonucleotides used for diagnostic PCRs and sequencings are not included.

| **Primer No.** | **Description** | **Sequence** |
| --- | --- | --- |
| TS-280 | pSNW2 fwd | agtcgacctgcaggcatg |
| TS-281 | pSNW2 rev | acagattaccctgttatccctatactg |
| TS-282 | fwd TS1 gtsA KT2440 | gggataacagggtaatctgttgggcgcggttgctgttg |
| TS-283 | rev TS1 gtsA KT2440 | tgactactttcagaactcatcggagcacctttcttgttgttatgc |
| TS-284 | fwd glf | atgagttctgaaagtagtcaggg |
| TS-285 | rew glf | ttacttctgggagcgccac |
| TS-286 | fwd TS2 gtsD in KT2440 | tgtggcgctcccagaagtaaaggacaacgtggctcacttc |
| TS-287 | rev TS2 gtsD in KT2440 | tgcatgcctgcaggtcgactgaagtcgcaagggaagctg |
| TS-288 | fwd TS1 gtsA PVLB_20095 | gggataacagggtaatctgtcgacctcaaccaggtgttg |
| TS-289 | rev TS1 gtsA PVLB_20095 | tgactactttcagaactcatgagagcaccttttcttgttg |
| TS-290 | fwd TS2 gtsD PVLB_20080 | tgtggcgctcccagaagtaaaggacaacgtggcccgct |
| TS-291 | rev TS2 gtsD PVLB_20080 | tgcatgcctgcaggtcgactacgggaagctgttgaagtcctc |
| TS-304 | fwd TS2 ex prom of 3303 in KT2440 | aggatcgtttctaatgactcacaacgttaatcaaaag |
| TS-305 | rev TS2 ex prom of 3303 in KT2440 | aagcttgcatgcctgcaggtcgacttcgaagccatagcgtatg |
| TS-307 | rev J23108 cloning | gttgtgagtcattagaaacgatcctccgcatg |
| TS-309 | fwd J23108 cloning in VLB landing pad | atcgctgaataatctagggcggcggatttg |
| TS-310 | fwd TS1 ex prom 3303 in LP VLB120 | gtatagggataacagggtaatctgtatgaagaaagacccgcgtg |
| TS-311 | rev TS1 ex prom 3303 in LP VLB120 | ccgccgccctagattattcagcgatcagccag |
| TS-329 | fwd flank1 landing Pad PP_1738 | gggataacagggtaatctgtagctggtgggcgatgacag |
| TS-330 | rev flank1 landing Pad PP_1738 | gatagaggtccaaatgccaccgcagcgg |
| TS-331 | fwd insert Pgts-Zm_glf (for KT2440) | gtggcatttggacctctatcacgcctac |
| TS-332 | rev insert Pgts-Zm_glf (for KT2440) | gctcgaattcgtagacgagtcaacggcc |
| TS-333 | fwd insert terminator in landing pad | actcgtctacgaattcgagctcggtacc |
| TS-334 | rev insert terminator in landing pad | gcaagtacgctatctgacgtccttggac |
| TS-335 | fwd flank2 landing Pad PP_1738 | acgtcagatagcgtacttgctatctgcaac |
| TS-336 | rev flank2 landing Pad PP_1738 | tgcatgcctgcaggtcgacttgctcaatttctgaaagctg |
| TS-337 | fwd flank1 landing Pad PVLB_06360-65 | gggataacagggtaatctgttggtgggcgacgacagttg |
| TS-338 | rev flank1 landing Pad PVLB_06360-65 | cggccccacgcgacaaagaccaccgcac |
| TS-339 | fwd insert Pgts-Zm_glf (for VLB) | gtctttgtcgcgtggggccgattgactg |
| TS-340 | rev insert Pgts-Zm_glf (for VLB) | gctcgaattcgtagacgtttcaacgtcccttg |
| TS-341 | fwd insert terminator in landing pad | aaacgtctacgaattcgagctcggtacc |
| TS-342 | rev insert terminator in landing pad | acatctggggtatctgacgtccttggac |
| TS-343 | fwd flank2 landing Pad PVLB_06360-65 | acgtcagataccccagatgttatggcgatg |
| TS-344 | rev flank2 landing Pad PVLB_06360-65 | tgcatgcctgcaggtcgactttcaacccttgcgcatctc |
| TS-368 | fwd FRT-Km-14f-stilbene module | gcgttaataaagaattcgagctcggtac |
| TS-369 | rev FRT-Km-14f-stilbene module | ctcgaattctttattaacgcggttcacg |

## **Table S4** Coding DNA fragments

| **Name** | **Sequence (5’ → 3’)** | **Note** |
| --- | --- | --- |
| Glf_Zm_ | ATGAGTTCTGAAAGTAGTCAGGGTCTAGTCACGCGACTAGCCCTAATCGCTGCTATAGGCGGCTTGCTTTTCGGTTACGATTCAGCGGTTATCGCTGCAATCGGTACACCGGTTGATATCCATTTTATTGCCCCTCGTCACCTGTCTGCTACGGCTGCGGCTTCCCTTTCTGGGATGGTCGTTGTTGCTGTTTTGGTCGGTTGTGTTACCGGTTCTTTGCTGTCTGGCTGGATTGGTATTCGCTTCGGTCGTCGCGGCGGATTGTTGATGAGTTCCATTTGTTTCGTCGCCGCCGGTTTTGGTGCTGCGTTAACCGAAAAATTATTTGGAACCGGTGGTTCGGCTTTACAAATTTTTTGCTTTTTCCGGTTTCTTGCCGGTTTAGGTATCGGTGTCGTTTCAACCTTGACCCCAACCTATATTGCTGAAATTGCTCCGCCAGACAAACGTGGTCAGATGGTTTCTGGTCAGCAGATGGCCATTGTGACGGGTGCTTTAACCGGTTATATCTTTACCTGGTTACTGGCTCATTTCGGTTCTATCGATTGGGTTAATGCCAGTGGTTGGTGCTGGTCTCCGGCTTCAGAAGGCCTGATCGGTATTGCCTTCTTATTGCTGCTGTTAACCGCACCGGATACGCCGCATTGGTTGGTGATGAAGGGACGTCATTCCGAGGCTAGCAAAATCCTTGCTCGTCTGGAACCGCAAGCCGATCCTAATCTGACGATTCAAAAGATTAAAGCTGGCTTTGATAAAGCCATGGACAAAAGCAGCGCAGGTTTGTTTGCTTTTGGTATCACCGTTGTTTTTGCCGGGGTATCCGTTGCTGCCTTCCAGCAGTTGGTCGGTATTAACGCCGTGCTGTATTATGCACCGCAGATGTTCCAGAATTTAGGTTTTGGAGCTGATACGGCATTATTGCAGACCATCTCTATCGGTGTTGTGAACTTCATCTTCACCATGATTGCTTCCCGTGTTGTTGACCGCTTCGGCCGTAAACCTCTGCTTATTTGGGGTGCTCTCGGTATGGCTGCAATGATGGCTGTTTTAGGCTGCTGTTTCTGGTTCAAAGTCGGTGGTGTTTTGCCTTTGGCTTCTGTGCTTCTTTATATTGCAGTCTTTGGCATGTCATGGGGCCCTGTCTGCTGGGTTGTTCTGTCAGAAATGTTCCCGAGTTCCATCAAGGGCGCAGCTATGCCTATCGCTGTTACCGGACAATGGTTAGCTAATATCTTGGTTAACTTCCTGTTTAAGGTTGCTGATGGTTCTCCAGCATTGAATCAGACTTTCAACCACGGTTTCTCCTATCTCGTTTTCGCAGCATTAAGTATCTTAGGTGGCTTGATTGTTGCTCGCTTCGTGCCGGAAACCAAAGGTCGGAGCCTGGATGAAATCGAGGAGATGTGGCGCTCCCAGAAGTAA | Coding sequence for glucose facilitator protein (UniProt P21906)  from *Zymomonas mobilis* |

## **Figure S5**:

Growth of *P. taiwanensis* VLB120 GRC3, GRC3Δ*gcd* and GRC3Δ6MC‑III with either replaced glucose transporter gene *gtsABCD* by *Zm_glf* or with *Zm_glf* expression from landing pad PVLB_06360/65. Grown in Growth Profiler with 20 mM glucose and 3-fold buffered MSM for 25 h with inoculation to OD_600_ 0.1 from adaption cultures in MSM. Error bars represent the standard deviation (n=4). Conversion to ‘OD_600_ equivalent’ by equation OD_600_ = a*(gvalue-gblanc)^b + c*(gvalue-gblanc)^d + e*(gvalue-gblank)^f (a, 0.0267; b, 1.01; c, 0.00000399; d, 3.18, e, 0.00000000000004; f, 0.01).


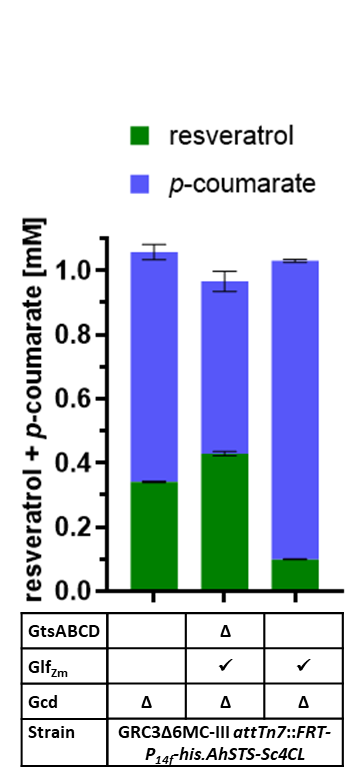


## **Figure S6**:

Stacked concentrations of *p*-coumarate and resveratrol of GRC3Δ6MC‑III with stilbene module (*attTn7*::*FRT-P_14f_-his.AhSTS-AtPAL2*) with either replaced glucose transporter gene *gtsABCD* by *Zm_glf* or with *Zm_glf* expression from landing pad PVLB_06360/65. Grown in 24-square deep well plate with 30 mM (5.4 g L^-1^) glucose, 3-fold buffered MSM and 1 mM *p*-coumarate for 24 h. Error bars represent the standard deviation (n=3). Same data as Fig. 4 in the main article.

# References

Ackermann, Y. S., Li, W.-J., Op de Hipt, L., Niehoff, P.-J., Casey, W., Polen, T., Köbbing, S., Ballerstedt, H., Wynands, B., O’Connor, K., Blank, L. M., & Wierckx, N. (2021). Engineering adipic acid metabolism in *Pseudomonas putida*. *Metabolic Engineering*, *67*(February), 29–40. https://doi.org/10.1016/j.ymben.2021.05.001

Choi, K. H., Gaynor, J. B., White, K. G., Lopez, C., Bosio, C. M., Karkhoff-Schweizer, R. A. R., & Schweizer, H. P. (2005). A Tn7-based broad-range bacterial cloning and expression system. *Nature Methods*, *2*(6), 443–448. https://doi.org/10.1038/nmeth765

De Las Heras, A., Carreño, C. A., & De Lorenzo, V. (2008). Stable implantation of orthogonal sensor circuits in Gram-negative bacteria for environmental release. *Environmental Microbiology*, *10*(12), 3305–3316. https://doi.org/10.1111/j.1462-2920.2008.01722.x

Ditta, G., Stanfield, S., Corbin, D., & Helinski, D. R. (1980). Broad host range DNA cloning system for Gram-negative bacteria: Construction of a gene bank of *Rhizobium meliloti*. *Proceedings of the National Academy of Sciences of the United States of America*, *77*(12 II), 7347–7351. https://doi.org/10.1073/pnas.77.12.7347

Martínez-García, E., & de Lorenzo, V. (2011). Engineering multiple genomic deletions in Gram-negative bacteria: Analysis of the multi-resistant antibiotic profile of *Pseudomonas putida* KT2440. In *Environmental Microbiology* (Vol. 13, Issue 10, pp. 2702–2716). https://doi.org/10.1111/j.1462-2920.2011.02538.x

Otto, M., Wynands, B., Lenzen, C., Filbig, M., Blank, L. M., & Wierckx, N. (2019). Rational Engineering of Phenylalanine Accumulation in *Pseudomonas taiwanensis* to Enable High-Yield Production of *Trans*-cinnamate. *Frontiers in Bioengineering and Biotechnology*, *7*(312). https://doi.org/10.3389/fbioe.2019.00312

Schwanemann, T., Otto, M., Wynands, B., Marienhagen, J., & Wierckx, N. (2023). A *Pseudomonas taiwanensis* malonyl-CoA platform strain for polyketide synthesis. *Metabolic Engineering*, *77*(February), 219–230. https://doi.org/10.1016/j.ymben.2023.04.001

Volke, D. C., Friis, L., Wirth, N. T., Turlin, J., & Nikel, P. I. (2020). Synthetic control of plasmid replication enables target- and self-curing of vectors and expedites genome engineering of *Pseudomonas putida*. *Metabolic Engineering Communications*, *10*(January), e00126. https://doi.org/10.1016/j.mec.2020.e00126

Volke, D. C., Wirth, N. T., & Nikel, P. I. (2021). Rapid Genome Engineering of *Pseudomonas* Assisted by Fluorescent Markers and Tractable Curing of Plasmids. *Bio-Protocol*, *11*(4), e3917. https://doi.org/10.21769/BioProtoc.3917

Wirth, N. T., Kozaeva, E., & Nikel, P. I. (2020). Accelerated genome engineering of *Pseudomonas putida* by I‐*Sce*I―mediated recombination and CRISPR‐Cas9 counterselection. *Microbial Biotechnology*, *13*(1), 233–249. https://doi.org/10.1111/1751-7915.13396

Wynands, B., Otto, M., Runge, N., Preckel, S., Polen, T., Blank, L. M., & Wierckx, N. (2019). Streamlined *Pseudomonas taiwanensis* VLB120 Chassis Strains with Improved Bioprocess Features. *ACS Synthetic Biology*, *8*(9), 2036–2050. https://doi.org/10.1021/acssynbio.9b00108
